# Identification of potential target genes of honokiol in overcoming breast cancer resistance to tamoxifen

**DOI:** 10.3389/fonc.2022.1019025

**Published:** 2022-12-19

**Authors:** Adam Hermawan, Herwandhani Putri, Naufa Hanif, Nurul Fatimah, Heri Himawan Prasetio

**Affiliations:** ^1^ Laboratory of Macromolecular Engineering, Department of Pharmaceutical Chemistry, Faculty of Pharmacy, Universitas Gadjah Mada Sekip Utara II, Yogyakarta, Indonesia; ^2^ Cancer Chemoprevention Research Center, Faculty of Pharmacy, Universitas Gadjah Mada Sekip Utara II, Yogyakarta, Indonesia; ^3^ Laboratory of Advanced Pharmaceutical Sciences, Faculty of Pharmacy, Universitas Gadjah Mada Sekip Utara II, Yogyakarta, Indonesia

**Keywords:** honokiol, tamoxifen resistance, breast cancer, bioinformatics, targeted therapy

## Abstract

**Background:**

Honokiol (HON) inhibits epidermal growth factor receptor (EGFR) signaling and increases the activity of erlotinib, an EGFR inhibitor, in human head and neck cancers. In this study, using a bioinformatics approach and *in vitro* experiments, we assessed the target genes of HON against breast cancer resistance to tamoxifen (TAM).

**Materials and methods:**

Microarray data were obtained from GSE67916 and GSE85871 datasets to identify differentially expressed genes (DEGs). DEGs common between HON-treated and TAM-resistant cells were analyzed by Gene Ontology (GO) and Kyoto Encyclopedia of Genes and Genomes (KEGG) pathway enrichment analyses and protein-protein interaction (PPI) networks were constructed. Selected genes were analyzed for genetic alterations, expression, prognostic value, and receiver operating characteristics (ROC). TAM-resistant MCF-7 (MCF-7 TAM-R) cells were generated and characterized for their resistance toward TAM. A combination of HON and TAM was used for cytotoxicity and gene expression analyses. Molecular docking was performed using the Molecular Operating Environment software.

**Results:**

PPI network analysis revealed that *FN1*, *FGFR2*, and *RET* were the top three genes with the highest scores. A genetic alteration study of potential target genes revealed *MMP16* and *ERBB4* as the genes with the highest alterations among the breast cancer samples. Pathway enrichment analysis of *FGFR2, RET, ERBB4, SOX2, FN1*, and *MMP16* showed that the genetic alterations herein were likely to impact the RTK-Ras pathway. The expression levels of *RET*, *MMP16*, and *SOX2* were strongly correlated with prognostic power, with areas under the ROC curves (AUC) ​​of 1, 0.8, and 0.8, respectively. The HON and TAM combination increased TAM cytotoxicity in MCF-7 TAM-R cells by regulating the expression of potential target genes *ret*, *ERBB4*, *SOX2*, and *FN1*, as well as the TAM resistance regulatory genes including *HES1*, *VIM*, *PCNA*, *TP53*, and *CASP7*. Molecular docking results indicated that HON tended to bind RET, ErbB4, and the receptor protein Notch1 ankyrin domain more robustly than its native ligand.

**Conclusion:**

HON could overcome breast cancer resistance to TAM, potentially by targeting *FGFR2*, *RET*, *ERBB4*, *MMP16*, *FN1*, and *SOX2*. However, further studies are required to validate these results.

## Introduction

Tamoxifen (TAM), a selective estrogen receptor modulator, is the most commonly used drug in the estrogen receptor (ER)-positive breast cancer treatment (1). The effectiveness of TAM therapy in breast cancer decreases because of the drug resistance (2). The mechanisms of TAM resistance have been widely studied, including the downregulation of ER-alpha (3) and ligand-independent signaling activation (4). Ligand-independent signaling activation can occur due to the crosstalk with epidermal growth factor receptor (EGFR), for example, crosstalk between EGFR, insulin-like growth factor receptor type 1, and human epidermal growth factor receptor 2 (HER2) (5). Therefore, the inhibition of EGFR and HER2 signaling is a strategic approach to overcoming TAM resistance.

**Figure 1:**
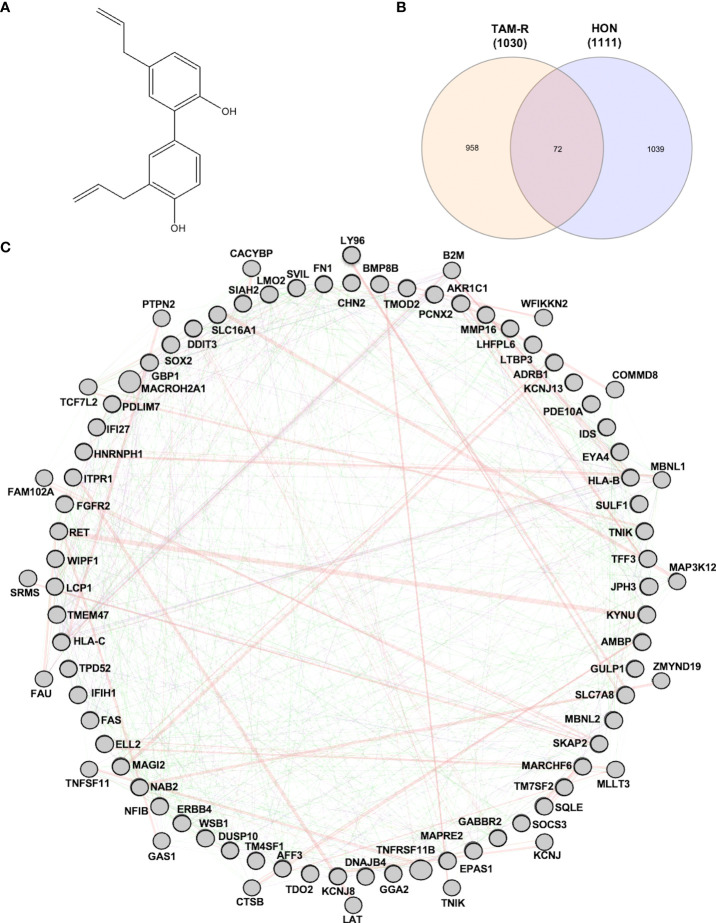
**(A)** Structure of honokiol (HON). **(B)** Venn diagram of differentially expressed genes (DEGs) from tamoxifen-resistant (TAM-R) and HON-treated MCF-7 cells. **(C)** Protein-protein interaction network of the protein related to overcoming TAM resistance by HON as analyzed using GeneMANIA.

Honokiol (3,5-di-2-propenyl-1,1-biphenyl-2,4-diol, HON) (**Figure 1A**) is a lignan isolated from the Magnolia plant species (6). HON has been widely studied as an anticancer treatment for breast cancer (7) and head and neck squamous cell carcinoma (8). Toxicity studies have shown that HON has a high safety profile and no reported adverse effects (9). A review article that discussed the anticancer activity of HON both *in vitro* and *in vivo* showed that HON inhibits the proliferation, invasion, and migration of cancer cells. Moreover, the molecular targets of HON in cancer cells include NF-κB, mTOR, EGFR, BMP7, STAT3, and hedgehog (10).

HON has also been used in combination with other anticancer agents. HON increased the cytotoxicity of bleomycin in MCF-7, PANC1, and UACC903 cells (11). The combination of HON and doxorubicin exerted a synergistic effect on doxorubicin-resistant breast cancer cell lines by inducing apoptosis (7). Furthermore, HON had cytotoxic effects on the ER+ TAM-resistant breast cancer cells having HER2 overexpression (7). One of the mechanisms of TAM resistance in breast cancer cells involves crosstalk with the human EGFR (2). In head and neck cancer, HON inhibits the EGFR signaling and increases the activity of the EGFR inhibitor erlotinib (8). Therefore, we hypothesized that HON could overcome TAM resistance in breast cancer cells by targeting EGFR signaling.

This study aimed to explore the potential downstream targets and mechanisms of HON’s action in circumventing breast cancer resistance to TAM using a bioinformatics approach.

## Materials and methods

### Data mining

Microarray data of MCF-7 TAM-R cells were obtained from GSE67916 (12). It contains the two cell lines, the TAM-R and TAM-sensitive MCF-7 cells. Microarray data of HON-treated MCF-7 cells were obtained from GSE85871 (13). Data processing was conducted using GEO2R, an online tool for GEO data analysis, based on the R programming language (https://www.ncbi.nlm.nih.gov/geo/geo2r/). DEGs between TAM-sensitive and TAM-resistant cells, and HON-treated and control were screened. The adjusted *p-value* < 0.05 and log |Fold Change| > 2 were used to select significant DEGs.

### GO and KEGG pathway enrichment analyses

GO and KEGG pathway enrichment analyses were conducted using DAVID v6.7 (14). A *p* < 0.05 was considered the cutoff value.

### Construction of PPI network and hub gene selection

The PPI network was visualized using GENEMANIA and default parameters (https://genemania.org/). Genes having the highest degree score of 5, analyzed using the cytoHubba plugin, were selected as hub genes (15, 16).

### Genetic alterations in the potential target genes

The genetic alterations in selected genes were analyzed using cBioPortal (http://www.cbioportal.org) (17, 18). In this study, the protein-coding genes, *FGFR2*, *RET, ERBB4*, *SOX2*, *FN1*, and *MMP16* were screened for genetic alterations in all the breast cancer studies available in the cBioPortal database. The breast cancer study with the highest genetic alterations was chosen for further oncoprinting, copy number alterations, gene network connectivity, pathways related to genetic alterations, and mutual exclusivity analysis. A *p* < 0.05 was considered the cutoff for mutual exclusivity analysis. Statistical analysis was conducted using one-way ANOVA with Tukey’s multiple comparison test, and Student’s *t*-test was used to analyze copy number alterations. *p < 0.05, **p < 0.01, ***p < 0.001, and ****p < 0.001.

### Gene expression analysis

Gene expression analysis of *FGFR2, ERBB4, RET, FN1, MMP16*, and *SOX2* in breast cancer patients from the TCGA study was performed using Gene Expression Profiling Interactive Analysis (GEPIA) (http://gepia2.cancer-pku.cn) (19). Briefly, gene symbols were submitted to GEPIA and analyzed in tumor vs. normal tissues. Gene expression was assessed in stage I–IV breast cancer samples. Statistical significance was set at *p* < 0.01.

### Prognostic value

Prognostic values related to *FGFR2, ERBB4, RET, FN1, MMP16*, and *SOX2* were analyzed using the KMPlotter of breast cancer patients (https://kmplot.com) (20). Briefly, gene symbols were submitted to KMPlotter, and several parameters were selected, including ER status positive, relapse-free survival (RFS), overall survival (OS), and patients receiving endocrine therapy with TAM only. Statistical significance was set at *p* < 0.05.

### ROC Plot

The correlation between gene expression and sensitivity to TAM in breast cancer patients was analyzed using the ROC plotter (http://www.rocplot.org) (21). Briefly, gene symbols were submitted to the ROC plotter, and several parameters were selected, including ER status positive, RFS at five years, OS, and patients receiving endocrine therapy with TAM. Statistical significance was set at p < 0.05.

### Cell culture

MCF-7 cells (ATCC) were cultured in Dulbecco’s Modified Eagle Medium-high glucose (Gibco) containing 20% of fetal bovine serum (FBS; Gibco), L-glutamine (Gibco), and penicillin-streptomycin, and kept at 37 °C in a 5% CO_2_ incubator. MCF-7 TAM-R cells were prepared as previously described (22). Briefly, MCF-7 cells were treated with 10 µM TAM for 72 h. The cells were cultured in a fresh medium and maintained until recovery. After recovery, the cells retreated with 10 µM TAM, and the previous steps were repeated seven times.

### Cytotoxicity

Cells were seeded (3,000 cells/well) in a 96-well plate and incubated until they reached 80% confluency. TAM (purchased from Sigma), HON (HON, purchased from Sigma), or a combination of both was added to the wells and incubated for 72 h. After incubation, MTT solution was added and cells were reincubated for 3 h. The resulting formazan crystals were dissolved in DMSO. The cell viability was calculated to be 5 for the control. We calculated the IC50 value with GraphPad Prism 5.0 using non-linear regression (curve fit) log (agonist) vs. normalized response-variable slope. Moreover, statistical analyses for the cytotoxicity data of TAM alone in MCF-7 and MCF-7 TAM-R, and HON alone and in combination in MCF-7 TAM-R, were conducted using two-way ANOVA with Sidak’s multiple comparison test. *p < 0.05, **p < 0.01, ***p < 0.001, and ****p < 0.001.

### Quantitative real-time polymerase chain reaction (qRT-PCR)

Cells were cultured, seeded (3 × 10^6^ cells in a 10-cm plate), incubated until 80% confluency, and treated with 10 μM of TAM, 10 μM of HON, a combination of TAM and HON, or DMSO for 72 h. Next, the cells were lysed and RNA was extracted using a QIAGEN RNA isolation kit. RNA was then transcribed into cDNA using a SensiFAST cDNA synthesis kit (Meridian Bioscience). Gene expression was measured in Bio-Rad CFX using the SensiFAST SYBR^®^ No-ROX Kit (Meridian Biosciences) with the selected primers (**Supplementary Table 1**). Gene expression was analyzed using the comparative threshold cycle (ΔΔCT). Statistical analyses of the MCF-7 vs. MCF-7 TAM-R cells were conducted using the Student’s *t-*test. Statistical analyses of the combination treatments were conducted using one-way ANOVA with Tukey’s multiple comparison test. *p < 0.05, **p < 0.01, ***p < 0.001, and ****p < 0.001.

### Molecular docking

To predict the binding characteristics between HON and RET, ErbB4, FGFR2, and the ankyrin domain of Notch1, molecular docking was conducted. The computational prediction was simulated on a Windows 10 operating system, Intel Core(TM) i5-10^th^ Gen processor with 8 GB of RAM. The software MOE 2010 (licensed by the Faculty of Pharmacy, UGM) was used for docking simulation, RMSD-docking score calculation, and visualization interaction. The PDB ID of the RET (2IVU), ErbB4 (3BBT), FGFR2 (2PVF), and the ankyrin domain of Notch1 (2HE0) was used for the search on rcsb.org. The chemical structure of HON and the natural ligand of the protein, were constructed using MarvinSketch. These structures were then subjected to conformational search and minimized in MOE using the energy-minimizing menu. For the docking simulation setting, London dG was used for both Rescoring 1 and Rescoring 2, Triangle Matcher was used for score function and placement setting, and Forcefield was used to refine the docking results from 30 retained settings. The results of this method helped determine which conformation has the lowest binding energy for interaction between the ligand and its receptor.

## Results

### Data mining

To explore the target genes of HON in circumventing breast cancer resistance to tamoxifen (TAM), we first used GSE67916, which contained microarray data from MCF-7 TAM-R cells and MCF-7 parental cells. We obtained DEGs and considered them to be regulatory genes of TAM-R in MCF-7 cells. We also used GSE85871, which contained microarray data from HON-treated and DMSO-treated MCF-7 cells to retrieve the DEGs of HON-treated MCF-7 cells to obtain the DEGs of HON-treated cells. A total of 1,030 and 1,111 genes were extracted from the GSE67916 (**Supplementary Table 2**) and GSE85871 (**Supplementary Table 3**) datasets, respectively. Moreover, a Venn diagram of DEGs from GSE67916 and GSE85871 was generated using InteractiVenn (http://www.interactivenn.net) (23), resulting in 72 overlapping DEGs (**Figure 1B** and **Supplementary Table 4**). Here we used 2 DEGs from 2 GSEs as a data mining model to search for target genes that regulate TAM resistance in MCF-7 cells, and also target HON in MCF-7 cells.

### GO and KEGG pathway enrichment analyses

The GO analysis was divided into three categories: biological processes, cellular components, and molecular functions. Several DEGs (**Table 1**) participated in the biological responses to steroid hormones, endogenous stimuli, toxins, nutrient levels, and extracellular stimuli. DEGs were located in the plasma membrane, cell fraction, vesicular fraction, and insoluble fraction. The DEGs also had a molecular function in transmembrane receptor protein tyrosine kinase activity and calcium ion binding. KEGG pathway enrichment analysis of the DEGs revealed their regulation of endocytic pathways (**Table 2**).

**Table 1:** GO enrichment analysis of the overlapping DEGs.

Term	*p* Value	Genes
**Biological Process**		
GO:0010033~response to organic substance	0.002976409	*TNFRSF11B, KYNU, ERBB4, SOCS3, KCNJ8, SQLE, TFF3, FAS, DNAJB4, DDIT3*
GO:0042592~homeostatic process	0.003903185	*JPH3, ADRB1, ERBB4, EPAS1, NAB2, SLC7A8, FAS, ITPR1, AKR1C1, DDIT3*
GO:0019442~tryptophan catabolic process to acetyl-CoA	0.008409523	TDO2, KYNU
GO:0031667~response to nutrient levels	0.009444695	TNFRSF11B, KYNU, SOCS3, SOX2, DDIT3
GO:0009991~response to extracellular stimulus	0.013726993	TNFRSF11B, KYNU, SOCS3, SOX2, DDIT3
GO:0048878~chemical homeostasis	0.020255432	JPH3, ERBB4, EPAS1, NAB2, SLC7A8, ITPR1, AKR1C1
GO:0007584~response to nutrient	0.021138651	*TNFRSF11B, KYNU, SOX2, DDIT3*
GO:0019441~tryptophan catabolic process to kynurenine	0.025020664	*TDO2, KYNU*
GO:0042436~indole derivative catabolic process	0.025020664	*TDO2, KYNU*
GO:0046218~indolalkylamine catabolic process	0.025020664	*TDO2, KYNU*
GO:0006569~tryptophan catabolic process	0.025020664	*TDO2, KYNU*
GO:0009636~response to toxin	0.027233825	*SLC7A8, FAS, AKR1C1*
GO:0009719~response to endogenous stimulus	0.027511399	*TNFRSF11B, ERBB4, SOCS3, TFF3, FAS, DDIT3*
GO:0006568~tryptophan metabolic process	0.029130546	*TDO2, KYNU*
GO:0048568~embryonic organ development	0.035837789	*LMO2, EPAS1, SOX2, DDIT3*
GO:0043129~surfactant homeostasis	0.041358337	*ERBB4, EPAS1*
GO:0048875~chemical homeostasis within a tissue	0.041358337	*ERBB4, EPAS1*
GO:0046700~heterocycle catabolic process	0.041728975	*AMBP, TDO2, KYNU*
GO:0001501~skeletal system development	0.045171114	*TNFRSF11B, PDLIM7, LTBP3, NAB2, BMP8B*
GO:0048545~response to steroid hormone stimulus	0.047113263	*TNFRSF11B, ERBB4, SOCS3, FAS*
GO:0009266~response to temperature stimulus	0.047765424	*ADRB1, SOCS3, DNAJB4*
GO:0009074~aromatic amino acid family catabolic process	0.049426007	*TDO2, KYNU*
GO:0048871~multicellular organismal homeostasis	0.049844689	*ADRB1, ERBB4, EPAS1*
**Cellular components**		
GO:0005792~microsome	0.002061103	*AMBP, JPH3, ADRB1, KCNJ8, SQLE, ITPR1*
GO:0000267~cell fraction	0.002220122	*JPH3, KYNU, MAGI2, HLA-C, HLA-B, ITPR1, AMBP, SLC16A1, TDO2, ADRB1, SQLE, KCNJ8, FAS*
GO:0042598~vesicular fraction	0.002339652	*AMBP, JPH3, ADRB1, KCNJ8, SQLE, ITPR1*
GO:0044459~plasma membrane part	0.006326136	*TM7SF2, MAGI2, ERBB4, SLC7A8, MMP16, HLA-C, HLA-B, GABBR2, KCNJ13, TMEM47, ADRB1, KCNJ8, SVIL, TM4SF1, FAS, LCP1, FN1, GBP1*
GO:0005624~membrane fraction	0.011112361	*AMBP, JPH3, SLC16A1, ADRB1, MAGI2, KCNJ8, SQLE, HLA-C, HLA-B, ITPR1*
GO:0005626~insoluble fraction	0.013643187	*AMBP, JPH3, SLC16A1, ADRB1, MAGI2, KCNJ8, SQLE, HLA-C, HLA-B, ITPR1*
**Molecular functions**		
GO:0004714~transmembrane receptor protein tyrosine kinase activity	0.034902686	FGFR2, RET, ERBB4
GO:0005509~calcium ion binding	0.046276593	RET, IDS, LTBP3, SVIL, SULF1, MMP16, TPD52, ITPR1, LCP1

**Table 2:** KEGG pathway enrichment analysis of the overlapping DEGs.

Term	*p* Value	Genes
hsa04144: Endocytosis	0.011566334	*FGFR2, RET, ADRB1, ERBB4, HLA-C, HLA-B*
hsa05200: Pathways in cancer	0.073530491	*FGFR2, RET, EPAS1, FAS, FN1*
hsa00100: Steroid biosynthesis	0.080493084	*TM7SF2, SQLE*

### PPI network construction and hub gene selection

A total of 72 genes were constructed in the PPI network complex with a confidence level of 0.4, containing 67 nodes and 40 edges, with an average node degree of 1.19, an average local clustering coefficient of 0.417, and a PPI enrichment *p-value* of 0.00288 (**Figure 1C**), which showed the complexity of the networks and highlighted that one protein interacted with another. Therefore, targeting several proteins in the PPI network is important for overcoming TAM resistance by HON. In order to reduce the complexity and select the most important protein in the PPI network, we performed hub gene selection based on the highest degree score. The top 10 genes with the highest degree scores were *FN1, SOX2, FGFR2, EPAS1, LMO1, LTBP3, MMP16, TNFRSF11B, AMBP*, and *RET* (**Table 3**).

**Table 3:** Top 10 hub genes based on degree score.

No.	Gene Symbol	Gene Name	Degree Score
1	*FN1*	Fibronectin type III domain-containing protein 1	10.5
2	*SOX2*	Transcription factor SOX2	9.25
3	*FGFR2*	Fibroblast growth factor receptor 2	8.25
4	*EPAS1*	Endothelial PAS domain-containing protein 1	7.94
5	*LMO2*	Rhombotin-2	7.9
6	*LTBP3*	Latent-transforming growth factor beta-binding protein 3	7.23
7	*MMP16*	Matrix metalloproteinase-16	6.48
8	*TNFRSF11B*	Tumor necrosis factor receptor superfamily member 11B	6.48
9	*AMBP*	Protein AMBP	6.48
10	*RET*	Proto-oncogene tyrosine-protein kinase receptor Ret	6.2

### Genetic alterations in *FGFR2, RET, ERBB4, SOX2, FN1*, and *MMP16*

Six target genes (*FGFR2, RET, ERBB4, SOX2, FN1*, and *MMP16)* were analyzed across breast cancer studies using the cBioPortal to assess genomic alterations. *FGFR2, RET*, and *ERBB4* were selected based on the KEGG pathway enrichment analysis results, which suggested the regulatory function of these genes in the endocytosis process. The genes, *FN1, SOX2*, and *MMP16*, were selected given their highest scores. *ERBB4* encodes HER4, a member of the tyrosine kinase receptor family, and a large membrane glycoprotein, playing a critical role in response to external stimuli (24). *FGFR2* encodes fibroblast growth factor receptor 2 (FGFR2), a member of the tyrosine kinase receptor family. *MMP16* encodes matrix metalloproteinase 16, a proteolytic enzyme that plays a role in extracellular matrix degradation during cancer metastasis (25). *FN1* encodes fibronectin 1, a glycoprotein that binds to receptors on the cell membrane and activates downstream signaling for cell survival, migration, invasion, and chemoresistance (26). *SOX2* is a key gene involved in the maintenance of stemness in embryonic and adult stem cells (27).

**Figure 2:**
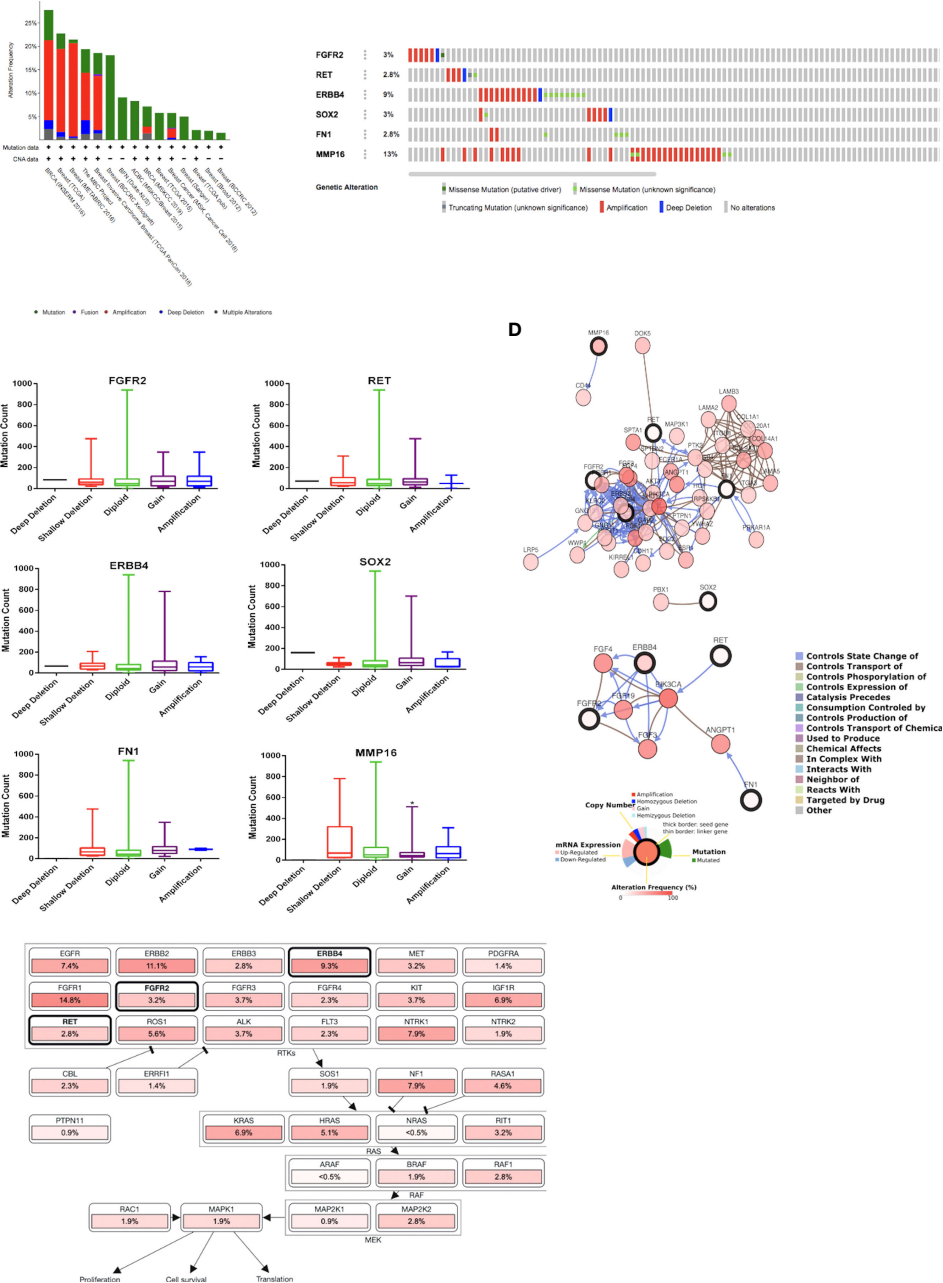
**(A)** Overview of changes in *FGFR2*, *RET*, *ERBB4*, *SOX2*, *FN1*, and *MMP16* in genomics dataset from 15 studies on breast cancer. **(B)** Summary of alterations of *FGFR2*, *RET*, *ERBB4*, *SOX2*, *FN1*, and *MMP16* across breast cancer samples [based on a study by Lefebvre et al. (2016)]. **(C)** Copy number of alterations of *FGFR2*, *RET*, *ERBB4*, *SOX2*, *FN1*, and *MMP16* across breast cancer samples [based on a study by Lefebvre et al. (2016)]. Statistical analysis was conducted using the one-way ANOVA with Tukey’s multiple comparison test. * indicates *p* < 0.05. **(D)** Gene network connected with *FGFR2*, *RET*, *ERBB4*, *SOX2*, *FN1*, and *MMP16* in breast cancer samples [based on a study by Lefebvre et al. (2016)]. **(E)** Pathway enrichment analysis related to the genetic alterations in *FGFR2*, *RET*, *ERBB4*, *SOX2*, *FN1*, and *MMP16* across breast cancer samples [based on a study by Lefebvre et al. (2016)].

The INSERM project (28) previously showed the highest genetic alterations among breast cancer studies, which were selected for further analysis (**Figure 2A**). Genetic alterations in each target gene were as follows: 2.8% (*RET*), 2.8% (*FN1*), 3% (*FGFR2*), 3% (SOX2), 9% (*ERBB4*), and 13% (*MMP16*) (**Figure 2B**). Moreover, most of the gene alterations were amplifications (**Figure 2B**). Further mutual exclusivity analysis showed that only one gene pair (*ERBB4–FN1*) exhibited significant co-occurrence (*p* < 0.05) in a breast cancer study of the INSERM project (**Table 4**). These results indicated the pivotal roles of *ERBB4* and *FN1* in HON treatment. Further analysis of copy number alterations revealed significant results. Copy number alteration analysis for *FGFR2, RET, ERBB4, SOX2, FN1*, and *MMP16* showed that only the mutation count of *MMP16* was significantly lower in cases with gain than in diploid or unchanged cases (**Figure 2C**). Analysis of the gene network associated with *FGFR2, RET, ERBB4, SOX2, FN1*, and *MMP16* suggested that *FGFR2, ERBB4*, and *FN1* may play important roles in this network (**Figure 2D**, upper part). *PIK3CA* and *FGF3* were found to be gene neighbors with the highest connectivity. To reduce network complexity, we screened neighbors according to 20% alterations, and the results yielded only four query genes, including *ERBB4, RET, FGFR2*, and *FN1* (**Figure 2D**, lower part). Pathway enrichment analysis of *FGFR2, RET, ERBB4, SOX2, FN1*, and *MMP16* showed that RTK-Ras is related to corresponding genetic alterations (**Figure 2E**).

**Table 4:** Mutual exclusivity analysis of selected genes in metastatic breast cancer study.

A	B	*p* Value	Log2 Odds Ratio	Tendency
*ERBB4*	*FN1*	0.011	> 3	Co-occurrence

### Gene expressions of *FGFR2, RET, ERBB4, SOX2, FN1*, and *MMP16* in breast cancer samples

**Figure 3:**
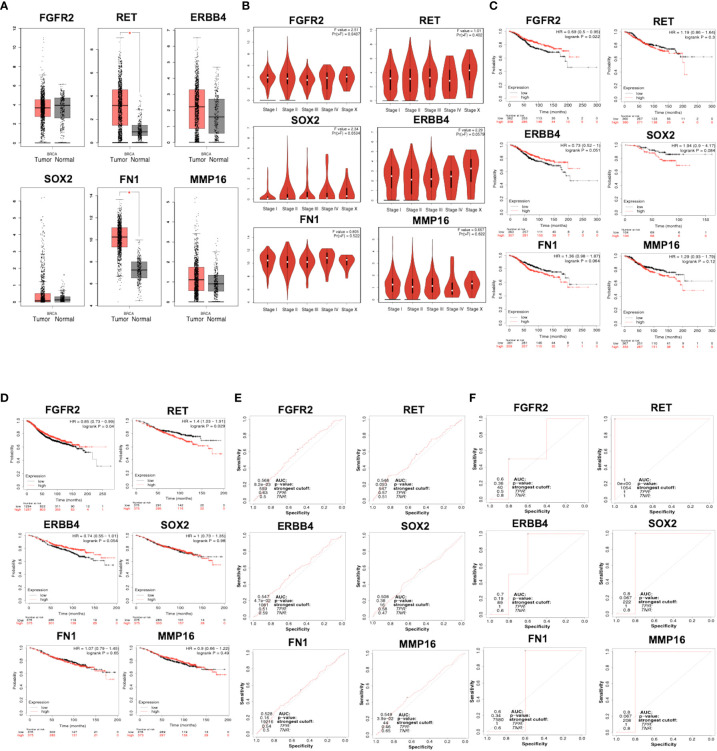
**(A)** mRNA expression of *FGFR2*, *ERBB4*, *RET*, *FN1*, *MMP16*, and *SOX2* in normal and breast cancer tissues, as analyzed by GEPIA. Breast tumor samples = 1085; normal tissues samples = 291. *p* < 0.01 was selected as a significant value. **(B)** The relationship between mRNA level and tumor stages in patients with breast cancer, as analyzed by GEPIA. *p* < 0.01 was selected as a significant value. **(C)** Overall survival related to the expression of *FGFR2*, *ERBB4*, *RET*, *FN1*, *MMP16*, and *SOX2*, as analyzed by KMPlotter. **(D)** Relapse-free survival related to the expression of *FGFR2*, *ERBB4*, *RET*, *FN1*, *MMP16*, and *SOX2*, as analyzed by KMPlotter. The ROC plotter of the correlation between FGFR2, *ERBB4*, *RET*, *FN1*, *MMP16*, and *SOX2* expression and TAM sensitivity, as analyzed by relapse-free survival **(E)** and pathological complete response **(F)**.

Using GEPIA, *RET* and *FN1* levels were found to be significantly higher in breast tumor tissues than in normal tissues (**Figure 3A**). We also analyzed the relationship between the mRNA levels of *FGFR2, ERBB4, RET, FN1, MMP16*, and *SOX2* and tumor stages in breast cancer using GEPIA (**Figure 3B**) and found that the levels of *FGFR2* were significantly upregulated in stage I and remained stable across stages II–X. The levels of *ERBB4* were upregulated in stage I, downregulated in stages I–II and upregulated in stages III–X. The mRNA levels of *SOX2*, *MMP16*, and *FN1* remained stable across stages I–X. The levels of *RET* mRNA were upregulated in stages I–II, downregulated in stages III–IV, and upregulated in stages IV–X.

### Prognostic value

The prognostic value of mRNA expressions of *FGFR2, ERBB4, RET, FN1, MMP16*, and *SOX2* was analyzed using two parameters, OS and RFS. Patients with breast cancer showing low levels of *FGFR2* and *ERBB4* had worse OS relative to the other groups (*p* = 0.022) (**Figure 3C**). Moreover, patients with breast cancer having low mRNA levels of *RET, MMP16, FN1*, and *SOX2* had better OS; however, the difference was not significant. RFS analysis suggested that patients with breast cancer having low mRNA levels of *FGFR2* had significantly worse RFS relative to the other groups (*p* = 0.04), whereas patients with high levels of *ERBB4* had a better RFS than the opposite group (*p* = 9.5 × 10^-5^) (**Figure 3D**). In addition, patients with breast cancer with low mRNA levels of *RET, MMP16, FN1*, and *SOX2* had better RFS, but this was not significant in the opposite groups (*p* > 0.05).

### ROC plot shows strong prognostic power for *RET, MMP16*, and *SOX2* expression

The correlation between gene expression levels with TAM response according to RFS and pathological complete response (PCR) based on transcriptome data from patients with breast cancer was analyzed. The expression levels of *FGFR2, ERBB4*, and *MMP16* were significantly moderately correlated with AUC values ​​of 0.568, 0.547, and 0.549, respectively (**Figure 3E**). The expression levels of *RET, FN1*, and *SOX2* were not correlated with the RFS of patients treated with TAM. Using the PCR parameter, the expression levels of *RET, MMP16*, and *SOX2* showed strong prognostic power, with AUC values ​​of 1, 0.8, and 0.8, respectively (**Figure 3F**).

### Generation and characterization of MCF-7 TAM-R cells

**Figure 4:**
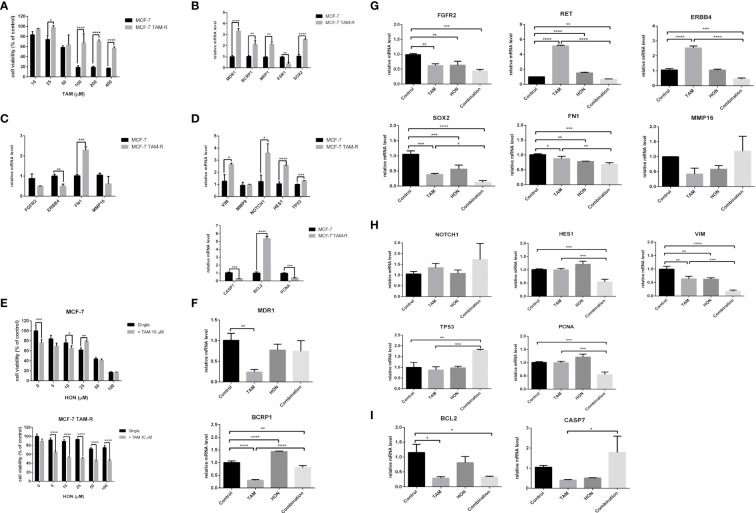
**(A)** Cytotoxicity of TAM in MCF-7 parental and MCF-7 TAM-R cells. Cells were seeded, incubated, and treated with a serial concentration of TAM. Cytotoxicity was determined using MTT assay and presented as cell viability as explained in the methods section. Results are shown as the average of three independent experiments (mean ± SD). Statistical analysis of the cytotoxicity of TAM single in MCF-7 and MCF-7 TAM-R cells was conducted using two-way ANOVA with Sidak’s multiple comparison test. *,**,***,**** indicates *p* < 0.05 or *p* < 0.01 or *p* < 0.001 or *p* < 0.0001, respectively. **(B)** mRNA expression of ABC transporter genes (*MDR1*, *BCRP1*, *MRP1*), *ESR1*, and *SOX2* in MCF-7 and MCF-7 TAM-R cells. **(C)** mRNA expression of potential target genes (*FGFR2*, *ERBB4*, *FN1*, and *MMP16*) in MCF-7 and MCF-7 TAM-R cells. **(D)** mRNA expression of apoptosis regulatory genes (*CASP7*, *BCL2*), *VIM*, *MMP9*, *NOTCH1*, *HES1*, *TP53*, and *PCNA* in MCF-7 and MCF-7 TAM-R cells. **(E)** HON increased the cytotoxicity of TAM in MCF-7 parental and MCF-7 TAM-R cells. Cells were seeded, incubated, and treated with a serial concentration of TAM. Cytotoxicity was determined using an MTT assay and presented as cell viability as explained in the methods section. Results are shown as the average of three independent experiments (mean ± SD). Statistical analyses of the HON single and in combination in MCF-7 TAM-R were conducted using two-way ANOVA with Sidak’s multiple comparison test. *,**,***,**** indicates *p* < 0.05 or *p* < 0.01 or *p* < 0.001 or *p* < 0.0001, respectively. **(F)** A combination of 10 μM of TAM and 10 μM of HON decreased the mRNA expression levels of ABC transporter gene *BCRP1*, but not *MDR1* in MCF-7 TAM-R cells. **(G)** The combination of TAM and HON decreased the mRNA expression levels of *RET*, *ERBB4*, *SOX2*, and *FN1*, but did not affect mRNA levels of *FGFR2* and *MMP16* in MCF-7 TAM-R cells. **(H)** The combination of 10 μM of TAM and 10 μM of HON decreased the mRNA expression levels of *HES1, VIM, PCNA*, and increased mRNA levels of *TP53* in MCF-7 TAM-R cells. **(I)** The combination of 10 μM of TAM and 10 μM of HON decreased the mRNA expression levels of BCL2, but increased mRNA levels of *CASP7* in MCF-7 TAM-R cells. Gene expression was calculated by q-RT PCR. GAPDH was used as an internal control. The results were analyzed using a comparative threshold cycle (ΔΔCT) and showed as a fold change relative to the control. Results are shown as the average of three independent experiments (mean ± SD). Statistical analyses of the mRNA in MCF-7 vs. MCF-7 TAM-R cells or HON, TAM, and combination-treated MCF-7 TAM-R cells were conducted using a student’s t-test. Statistical analyses of the mRNA upon combination treatment were conducted using one-way ANOVA with Tukey’s multiple comparison test. *,**,***,**** indicates *p* < 0.05 or *p* < 0.01 or *p* < 0.001 or *p* < 0.0001, respectively.

We successfully generated MCF-7 TAM-R cells by consecutive TAM treatment, as described in the methods section. MCF-7 TAM-R cells were more resistant towards TAM than the parental MCF-7 cells (**Figure 4A**). Moreover, TAM, at the concentrations of 25, 100, 200, and 400 μM, resulted in a significant increase in cell viability in MCF-7 TAM-R cells. The IC50 value of TAM in MCF-7 and MCF-7 TAM-R were 52.78 μM and 605.5 μM, respectively (or more than 400 μM, the highest concentration we used in the experiments). We then characterized the chemoresistance marker in TAM-R MCF-7 by qRT-PCR, and the results showed a significant increase in the mRNA levels of the ABC transporter genes *MDR1, BCRP1*, and *MRP1* (**Figure 4B**). We also observed a significant downregulation of *ESR1* and a significant upregulation of *SOX2* in MCF-7 TAM-R cells. Among the potential target genes, we observed a significant downregulation of *ERBB4* and a significant upregulation of *FN1* (**Figure 4C**). We further analyzed the expression of the downstream signaling genes of TAM resistance regulatory pathways, including *VIM, MMP9, NOTCH1, HES1*, and *TP53*, and found a significant upregulation of *VIM, NOTCH1, HES1*, and *TP53* in MCF-7 TAM-R cells (**Figure 4D**). In addition, the mRNA levels of pro-apoptosis regulatory genes *CASP7* were significantly decreased, the mRNA levels of the anti-apoptosis gene *BCL2* were significantly increased, and the expression levels of the proliferation marker *PCNA* were significantly decreased in MCF-7 TAM-R cells compared to that in the parental MCF-7 cells (**Figure 4D**).

### HON increased the sensitivity of TAM-R cells toward TAM

We evaluated the cytotoxicity of a series of concentrations of HON (0–100 μM) alone and in combination with 10 μM of TAM in both parental and MCF-7 TAM-R cells. The results revealed that the combination of TAM with HON at concentrations of 10 μM significantly increased the cytotoxicity compared to HON treatment alone in MCF-7 parental cells (**Figure 4E**, upper part). In addition, the combination of TAM with HON at concentrations of 5, 10, 25, 50, and 100 μM significantly increased the cytotoxicity compared to HON treatment alone, highlighting the importance of the HON-TAM combination in increasing TAM sensitivity in MCF-7 TAM-R cells (**Figure 4E**, lower part). The IC50 value in MCF-7 TAM-R was 600 μM for HON on its own and 32.13 μM for combined HON and TAM. The IC50 value in parental MCF-7 was 33.53 μM for HON on its own and 32.42 μM for combined HON and TAM. We then performed qRT-PCR to measure the mRNA levels upon treatment of HON, TAM, and combination in MCF-7 TAM-R cells. The combination of 10 μM TAM and 10 μM HON decreased the mRNA expression levels of the ABC transporter gene *BCRP1*, but not *MDR1* (**Figure 4F**). Moreover, the combination of TAM and HON decreased the mRNA expression levels of the potential target genes *RET*, *ERBB4*, *SOX2*, and *FN1* but did not affect the mRNA levels of *FGFR2* and *MMP16* (**Figure 4G**). We also examined the effect of the combination of 10 μM TAM and 10 μM HON on the TAM-R regulatory pathway, and observed a decrease in the mRNA expression levels of *HES1*, *VIM*, and *PCNA*, and increased mRNA levels of *TP53* (**Figure 4H**). The combination of 10 μM TAM and 10 μM HON also decreased the mRNA expression levels of anti-apoptotic *BCL2* but increased the mRNA levels of pro-apoptosis protein *CASP7* (**Figure 4I**).

### Molecular docking shows that HON inhibits RET, ErbB4, and Notch1

**Figure 5:**
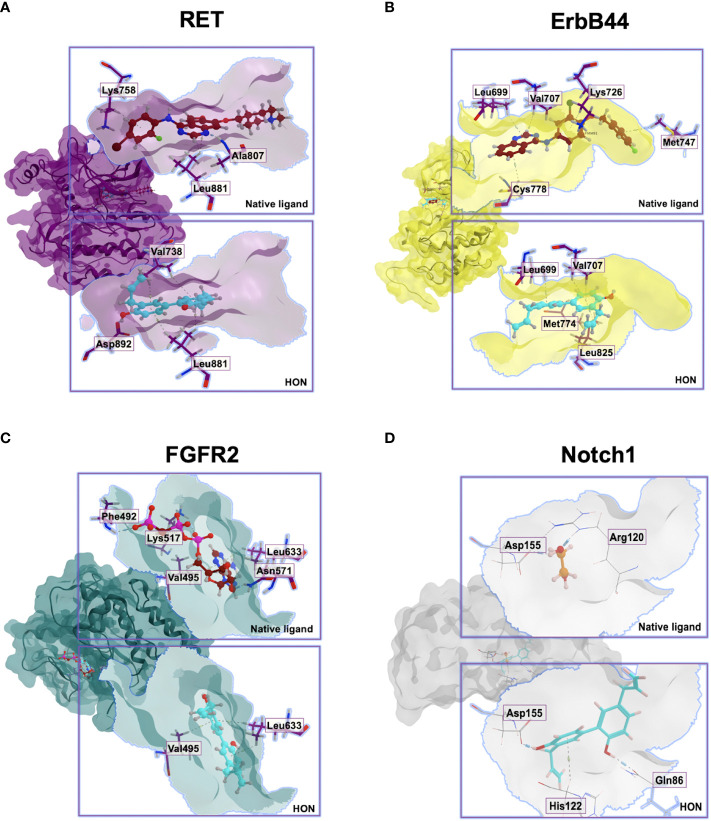
Molecular docking interactions between the **(A)**. RET, **(B)**. ErbB4, **(C)**. FGFR2, **(D)**. Ankyrin domain of Notch1, their native ligands, and HON.

Molecular docking studies for HON with RET, ErbB4, FGFR2, and Notch1 were performed. RET, ErbB4, and FGFR2 were selected from the results of the previous step. *FGFR2* was involved in the crosstalk with Notch signaling; therefore, we performed a molecular docking study of HON with Notch1. RET (PDB ID: 2IVU) docked with HON and its native ligand, vandetanib. ErbB4 (PDB ID: 3BBT) docked with HON and its native ligand, lapatinib. FGFR2 (PDB ID: 2PVF) docked with HON and its native ligand, phosphomethylphosphonic acid guanylate ester. The ankyrin domain of Notch1 (PDB ID: 2HE0) docked with HON and its native ligand, 1,2-ethanediol. An RMSD < 2 indicated the validity of the docking method (**Table 5**). HON had a slightly higher score than vandetanib. Three amino acids bound to HON and vandetanib, whereby Leu881 was the common amino acid (**Table 5** and **Figure 5A**). HON had a lower docking score than lapatinib; these results indicated that HON tended to bind to ErbB4 much better than lapatinib, even though only four amino acids were bound to HON as compared to five that were bound to lapatinib. Leu825 was the main amino acid bound to HON, stabilizing and making the binding between HON and ErbB4 stronger than that with lapatinib (**Table 5** and **Figure 5B**). HON had a higher docking score than phosphomethylphosphonic acid guanylate ester, and lower amino acid binding to FGFR2, with only two residues (**Table 5** and **Figure 5C**). HON had a lower score than 1,2-ethanediol, the native ligand of Notch1 (**Table 5**). This result indicated that HON tended to bind the receptor protein Notch1 ankyrin domain much better and in a more robust manner than 1,2-ethanediol. The amino acids that interacted with HON, including Gln86, His122, and Asp155, were more abundant than those of the native ligand, with only two amino acid residues (Arg120 and Asp155) (**Table 5** and **Figure 5D**).

**Table 5:** Molecular docking results of RET, ErbB4, FGFR2, ankyrin domain of NOTCH1, its native ligand, and Honokiol.

Protein (PDB ID)	Native Ligand						Honokiol					
	S	RMSD (Å)	LA	AA	BT	D	S	RMSD (Å)	LA	AA	BT	D
RET (2IVU)	-12.50	1.49	C N C	Lys758 Ala807 Leu881	ArH ScA ArH	3.94 1.95 3.79	-11.90	1.16	C C H	Val738 Leu881 Asp892	ArH ArH ScD	3.89 3.49 1.62
ErbB4 (3BBT)	-10.84	0.82	C C C C C	Leu699 Val707 Lys726 Met747 Cys778	ArH ArH ArH ArH ArH	3.95 4.24 4.27 4.05 4.13	-11.01	1.13	C C H C	Leu699 Val707 Met774 Leu825	ArH ArH ScD ArH	4.00 3.82 2.22 3.95
FGFR2 (2PVF)	-15.47	1.61	O^-^ N O^-^ O^-^ C	Phe492 Val495 Lys517 Asn571 Leu633	ScA ArH ScA ScA ArH	2.02 3.76 1.93 2.02 3.31	-10.82	1.76	C C	Val495 Leu633	ArH ArH	4.13 3.74
Notch1 (2HE0)	-5.56	1.54	O H	Arg120 Asp155	ScD ScA	2.89 2.06	-7.73	1.29	H C H	Gln86 His122 Asp155	ScA ArH ScA	2.11 4.31 2.03

S (docking score), RMSD (root mean square deviation), LA (ligand atom), AA (amino acid), BT (binding type), D (distance), ScD (sidechain donor), ScA (sidechain acceptor), ArH (arene H), BbD (backbone donor). Native ligands for RET, ErbB4, FGFR2, and Notch1 are Vandetanib, Lapatinib, Phosphomethylphosphonic acid guanylate ester, and 1,2-ethanediol, respectively.

## Discussion

Using a bioinformatics approach, this study explored the potential targets and mechanisms of HON in circumventing breast cancer resistance to TAM*. In vitro* experiments were used to validate the results of bioinformatics analysis. GO analysis showed that the DEGs, located in the plasma membrane fraction, were involved in the biological response to endogenous stimuli. Moreover, DEGs play a role in the molecular functions of transmembrane receptor protein tyrosine kinase activity and calcium ion binding. KEGG pathway enrichment analysis revealed the regulation of endocytosis by DEGs. Receptor-mediated endocytosis involves the uptake of molecules into cytoplasmic vesicles mediated by membrane receptors, including EGFRs (29). One of the hallmarks of cancer is the perturbation in the cycles of endocytosis, both trafficking and recycling EGFRs to the membrane (30). Therefore, targeting endocytosis can be a strategy for cancer therapy to deal with TAM resistance. HON interferes with the process of dengue virus endocytosis by abrogating the colocalization of viral glycoprotein envelopes and early endosomes (31). However, the role of HON in the endocytosis of TAM-resistant breast cancer cells remains unclear.

The PPI network and hub gene selection revealed the top three genes with the highest score degree, namely *FN1, FGFR2*, and *SOX2*. A genetic alteration study of the potential target genes revealed *MMP16* and *ERBB4* as the genes with the highest alterations among the breast cancer samples, with most of the alterations belonging to amplification. Mutual exclusivity analysis using cBioPortal revealed *ERBB4–FN1* was the only gene pair with significant co-occurrence. These results indicate that *ERBB4* and *FN1* are the key genes in HON treatment. Network analysis showed that *FGFR2*, *ERBB4*, and *FN1* were important players in the gene network. Moreover, when network complexity was reduced by 20%, four genes were revealed, namely, *FGFR2, ERBB4, FN1*, and *RET*.

We found that RTK-Ras is a pathway linked to genetic changes in *FGFR2, RET, ERBB4*, *SOX2*, *FN1*, and *MMP16*, according to the pathway enrichment analysis. Using GEPIA, we discovered that the levels of *RET* and *FN1* in tumor tissues were much higher than those in normal tissues. Patients with low *RET, MMP16, FN1*, and *SOX2* had a higher overall survival rate, but this was not statistically significant. The expression levels of *RET, MMP16*, and *SOX2* demonstrated substantial prognostic power associations, with AUC values of 1, 0.8, and 0.8, respectively. MCF-7 TAM-R cells were successfully produced, with increased mRNA levels of ABC transporter genes *MDR1, BCRP1, MRP1*, and *SOX2*, and considerable downregulation of *ESR1, ERBB4, CASP7*, and *PCNA*, as well as a large overexpression of *FN1*. Furthermore, we found that *VIM, NOTCH1, HES1, BCL2*, and *TP53* were significantly upregulated in MCF-7 TAM-R cells compared with MCF-7 parental cells.

In ER+ breast cancer cells, the effect of TAM is diminished by activation of the FGFR2 signaling (32). A point mutation in *FGFR2* has been detected in breast cancer cells (33). A study demonstrated that single-nucleotide polymorphisms in *FGFR2* reduce the expression of FGFR2 and increase cell sensitivity of breast cancer towards estrogen (34). The *RET* gene, a member of the tyrosine kinase receptor family, encodes for rearrangement during the transfection (35). The *RET* gene is overexpressed in ER+ breast cancer cells (36). Inhibition of RET signaling enhances TAM sensitivity in the ER+ breast cancer (37). The *ERBB4* gene encodes human EGFR4 (HER4), a tyrosine kinase receptor that regulates signaling pathways through the proteolytic release of intracellular and extracellular receptor fragments (38). *ERBB4* overexpression occurs in ER+ breast cancer cells (39). Taken together, *FGFR2, RET*, and *ERBB4* are potential targets of HON for overcoming ER+ breast cancer resistance to TAM.

MMP16 promotes the migration and invasion of cancer cells in the breast cancer (40). SOX2 is a cancer stem cell marker that is highly expressed in breast cancer stem cells and mediates resistance to chemotherapy (41). A previous study demonstrated that SOX2 reduces breast cancer cell sensitivity to TAM by activating Wnt signaling (42). Collectively, further studies targeting *MMP16, FN1*, and *SOX2* using HON will reveal the mechanism of overcoming TAM resistance.

FGFR2 and ERBB4 are human epidermal growth factor receptors, in which signaling is important for the maintenance of proliferation, survival, and stemness (43–46). FGFR signaling is known to cross-talk with Notch signaling. Notch signaling mediates FGFR signaling in the nephron progenitors (47). In addition, FGFR signaling stimulates radial glial identity and interacts with Notch1 signaling in the telencephalic progenitors (48). Moreover, FGFR2 signaling induces *SOX2* expression during the osteoblast differentiation (49). Overexpression of *SOX2* is correlated with FGFR fusion in human lung cancer cells (50). Inhibition of FGFR signaling leads to the downregulation of SOX2, which maintains the stemness of pancreatic cancer cells (51). A previous study demonstrated that HON inhibits Notch signaling in colon cancer stem cells (52) and melanoma stem cells (53). Therefore, future studies on the mechanism of HON targeting FGFR2 and Notch signaling in overcoming breast cancer resistance to TAM are needed.

Molecular docking results showed that HON can inhibit RET and ErbB4. HON had a slightly higher score than Vandetanib to bind with RET. Due to Leu825 stabilizing and making the binding between HON and ErbB4 stronger than Lapatinib, they have a higher tendency to bind together. This finding is supported by previous studies that demonstrated the binding of HON to the kinase domain of ErbB (54–56). Results of this study showed that HON has a higher docking score than Phosphomethylphosphonic acid guanylate ester to bind FGFR2; however, HON has been shown to inhibit FGFR signaling in lung squamous cell carcinoma (57). Therefore, further investigations on the effect of HON on FGFR2 are still needed. After validating the axis between SOX2, Notch, and FGFR2, we performed a molecular docking study to determine whether HON can act as a Notch inhibitor. Molecular docking results showed that HON is a potent inhibitor of the ankyrin domain of the Notch receptor. The ankyrin domain is an intracellular domain of the Notch receptor that plays a role in the Notch1 signaling (58). Ankyrins link membrane proteins to the cytoskeleton and function in protein expression and stability (59). The ankyrin domain in Notch plays a role in converting the transcriptional repression complex into an active complex (60). Ankyrin inhibition causes transcriptional repression of target genes in Notch1 signaling. HON was shown to inhibit the ankyrin domain of Notch1, as indicated by its lower binding energy than that of its native ligand (1,2-ethanediol). Results of the molecular docking study need to be confirmed using *in vitro* studies such as enzymatic assay, western blotting of the downstream signaling of RTK-Ras, and crystallographic study.

The results of this study showed modulation of gene expression in TAM-R MCF-7 cells. ERBB4 was downregulated while FN1 was upregulated in TAM-R cells. These findings are supported by previous studies that demonstrated the downregulation of *ERBB4* (61), and the upregulation of *FN1* in TAM resistance (62–64). Significant upregulation of *VIM, NOTCH1, HES1*, and *TP53* in was observed in TAM-R MCF-7 cells, indicating increased Notch signaling, epithelial to mesenchymal transition, and inhibition of cell cycle in TAM-R cells. The expression levels of the proliferation marker *PCNA* were significantly decreased in TAM-R MCF-7 cells, this result is supported by a study reported by Post (2020) which showed the downregulation of *PCNA* and genes involved in the cell cycle (65).

This study revealed that the cytotoxic effects of HON and TAM in TAM-R MCF-7 cells potentiated TAM efficacy. This effect is stronger in MCF-7 TAM-R than in MCF-7 parental cells, in which the combination of TAM and 25 μM of HON generated higher cell viability. Molecularly, the combination of HON and TAM decreased the mRNA expression levels of ABC transporter gene *BCRP1*, potential target genes, including *RET, ERBB4, SOX2, FN1*, regulatory genes of TAM-R, including *HES1, VIM, PCNA*, and *BCL2*; and increased the mRNA levels of *TP53* and *CASP7*. The combination of 10 μM TAM and 10 μM HON decreased the mRNA expression levels of the ABC transporter gene *BCRP1*, but not *MDR1.* In addition, TAM single treatment decreases the mRNA levels of *MDR1* and *BCRP1* in TAM-R cells. This finding is supported by data in previous studies that TAM treatment in colon cancer cells can reduce the expression level of *MDR1* (66). In addition, the decrease in *BCRP1* expression due to TAM treatment in MCF-7 TAM-R cells was also in accordance with a previous study by Selevar et al., 2011 which showed downregulation of *BCRP1* due to TAM in TAM-R cells (67). Furthermore, the increase in *BCRP1* expression in the HON treatment is probably because HON is a substrate of BCRP1, according to a previous study by (68). The same group also stated that HON was also able to inhibit the BCRP1 activity (68). In this study, we did not measure BCRP1 activity due to HON treatment, so although *BCRP1* mRNA increased, its activity may be decreased due to HON treatment. This topic becomes interesting for further research.

The combination of TAM and HON decreased the mRNA expression levels of the potential target genes *RET*, *ERBB4*, *SOX2*, and *FN1* but did not affect the mRNA levels of *FGFR2* and *MMP16.* The decrease in mRNA levels of RET was supported by a study by Plaza-Menacho (2010), in which the downregulation of *RET* increases the sensitivity of MCF-7 cells toward TAM (69). Downregulation of *ERBB4* is supported by a previous study that downregulation of ERBB leads to the increased sensitivity of breast cancer cells to TAM (70). Decrease expression of *FN1* and *SOX2* indicating the inhibition of migration, invasion, and maintenance of cancer stem cell stemness. We observed a decrease in the mRNA expression levels of *HES1*, *VIM*, and *PCNA*, and increased mRNA levels of *TP53*, indicating the inhibition of Notch signaling, EMT, and a decrease in cell cycle and DNA repair activity due to HON treatment.

**Figure 6:**
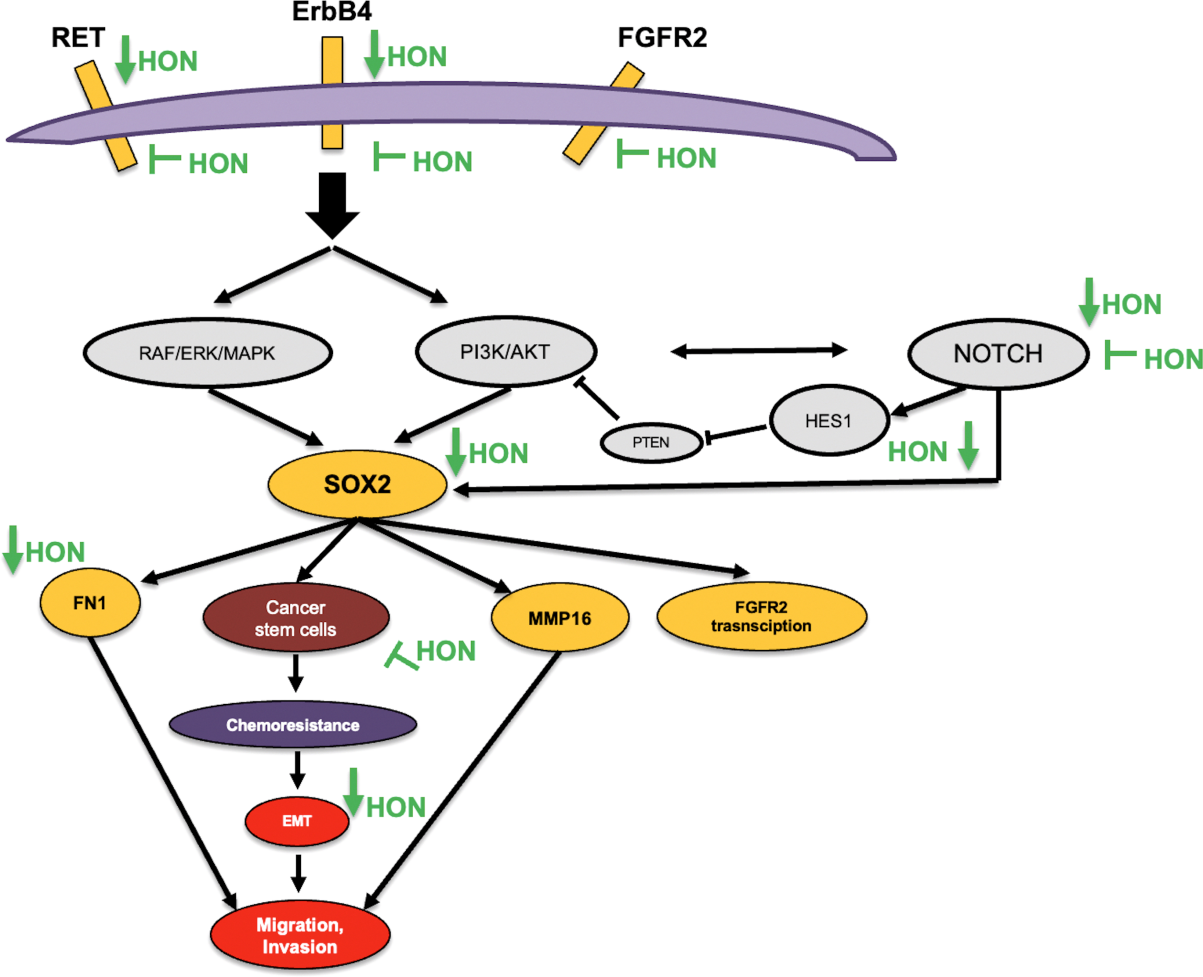
Proposed mechanism of HON in overcoming breast cancer resistance to TAM.

In this study, we postulated the mechanism of HON in overcoming breast cancer resistance towards TAM (**Figure 6**). Activation of ErbB and FGFR signaling leads to the activation of its downstream signaling, PI3K/Akt (71, 72) and the Ras/MAPK pathway (73). Activation of RET signaling promotes the activation of its downstream signaling Ras/MAPK and the PI3K/Akt pathways (74) Activation of Mapk signaling leads to the overexpression of SOX2 (75, 76) other studies demonstrated that activation of PI3k/Akt signaling was found to upregulate the expression of SOX-2 (77). The crosstalk between PI3K/Akt signaling and Notch was observed in the regulation of breast cancer development (78). In addition, Notch1 signaling regulates the expression of PTEN, an inhibitor of PI3K/AKT signaling, *via* HES1 (79). FN1 regulates the Notch signaling pathway (80). Another study showed that SOX2 is a transcription factor of FN1 that promotes the migration and invasion of ovarian cancer cells (81). Activation of FGFR2 signaling promotes downstream signaling PI3K/Akt and subsequently increases the expression level of MMP16 (82). A recent study demonstrated that SOX2 also promotes the expression of FGFR2 (75)

The ErbB signaling pathway is involved in the activation of alternative signaling pathways involved in TAM resistance (83). Moreover, upregulation of the receptor tyrosine kinase has been observed in TAM-R breast cancer cells (84). FGFR2 signaling plays a role in cancer-associated fibroblast-dependent breast cancer resistance to TAM (85). Downregulation of RET increased the sensitivity of MCF-7 breast cancer cells to TAM (69). Piva et al. (2014) showed that SOX2 is upregulated in TAM-R breast cancer cells and promotes breast cancer resistance to TAM (42). These results are also supported by those of Sommer et al. (2018), which demonstrated the upregulation of SOX2 in TAM-R breast cancer cells (22). Recently, SOX2 was shown to be a predictive marker for the early detection of TAM resistance in ER-positive breast cancer patients (86). Sox-2 promotes chemoresistance, maintains cancer stem cell properties, and induces epithelial-mesenchymal transition (87). The results of this study indicate that HON can inhibit RET, ErbB4, and Notch activity based on molecular docking studies. Moreover, the combination of HON and TAM reduced the expression of the potential target genes *SOX2*, *RET*, *ERBB4*, and *FN1*, as well as the neighboring genes *HES1*, *VIM*, and *PCNA*. This proves the potential of HON to overcome the resistance of breast cancer cells to TAM by inhibiting the expression of these target genes.

One limitation of this study is that the data mining was carried out indirectly on MCF-7 TAM-R cells treated with HON. However, this data mining model was further validated with MCF-7 TAM-R cells whose data are presented in our results. Another limitation is that the microarray data were derived from only one cell line. Therefore, it is necessary to conduct research using microarray data from other types of ER+ breast cancer cells such as T47D. Another limitation is that the molecular mechanism was studied at the mRNA level, and therefore, it needs further clarification at the protein level. This study used a bioinformatics approach to identify the potential target genes of HON and *in vitro* experiments to validate the bioinformatics findings *via* mRNA level measurements. Collectively, this study has accelerated the discovery of molecular targets and mechanisms of HON as a therapeutic agent to overcome the resistance of breast cancer cells to TAM. However, the results of this study need to be validated by measurement of protein levels *in vitro* as well as *in vivo* and by conducting clinical trials.

## Conclusions

In conclusion, *FGFR2*, *RET*, *ERBB4*, *MMP16*, *FN1*, and *SOX2* are potential targets of HON for overcoming TAM resistance in breast cancer. The combination of HON and TAM in TAM-R MCF-7 cells promoted TAM sensitivity. It also induced the downregulation of *BCRP1* and potential target genes, including *RET*, *ERBB4*, *SOX2*, *FN1*, *HES1*, *VIM*, *PCNA*, and *BCL2*, and elevated the mRNA levels of *TP53* and *CASP7*. In addition, molecular docking revealed that HON inhibits RET, ErbB4, and Notch signaling to overcome TAM resistance in breast cancer cells. However, further *in vitro* and *in vivo* and clinical studies are required to validate the results of this study.

## Data availability statement

The datasets presented in this study can be found in online repositories. The names of the repository/repositories and accession number(s) can be found in the article/**Supplementary Material**.

## Author contributions

AH conceptualized and designed the study; acquired, analyzed, and interpreted the data; and drafted and revised the article. HP and NH analyzed, and interpreted the data, and drafted the article, NF and HP acquired the data. All authors contributed to the article and approved the submitted version.
